# Effects of Endothelial and Mesenchymal Stem Cells on Improving Myocardial Function in a Sheep Animal Model

**Published:** 2017-04

**Authors:** Shahram Rabbani, Masoud Soleimani, Mohammad Sahebjam, Mohammad Imani, Seyed Mahdi Nassiri, Amir Atashi, Morteza Daliri Joupari, Ali Ghiaseddin, Mostafa Latifpour, Seyed Hossein Ahmadi Tafti

**Affiliations:** 1 *Tehran Heart Center, Tehran University of Medical Sciences, Tehran, Iran.*; 2 *Tarbiat Modares University, Tehran, Iran.*; 3 *Iran Polymer and Petrochemical Institute, Tehran, Iran.*; 4 *Faculty of Veterinary Medicine, University of Tehran, Tehran, Iran.*; 5 *Stem Cell and Tissue Engineering Research Center, Shahroud University of Medical Sciences, Shahroud, Iran.*; 6 *National Institute for Genetic Engineering and Biotechnology, Tehran, Iran.*

**Keywords:** *Endothelial cells*, *Mesenchymal stem cell transplantation*, *Myocardial infarction*, *Sheep*

## Abstract

**Background:** Myocardial infarction is the main cause of death worldwide. Angiogenesis, a promising new therapy for the treatment of diffuse coronary artery disease, shows a poor response to conventional revascularization techniques. This study focused on improving myocardial function using endothelial cells (ECs) and mesenchymal stem cells (MSCs) in a sheep animal model.

**Methods:** Acute myocardial infarction was induced in 18 sheep (12 treated cases and 6 controls). Autologous MSCs and ECs were injected in the infarcted area and the border zone. Two months after transplantation, echocardiography, electron microscopy, and immunohistochemistry were performed.

**Results:** Echocardiography in both MSC and EC groups revealed a significant improvement in the ejection fraction compared with the control group (p value < 0.05). Vascular density, estimated by antibodies against the von Willebrand factor and smooth muscle actin, increased in both study groups. The pattern of vascularity in the MSC and EC groups was diffused. The electron microscopic evaluation of the infracted areas revealed cardiomyocytes in variable stages of development in the border zone in both EC and MSC groups.

**Conclusion:** Both ECs and MSCs were able to promote angiogenesis and improve cardiac function. Presumably, MSCs differentiate into ECs and cause angiogenesis as it occurs for ECs.

## Introduction

Cardiovascular diseases are the main cause of hospitalization and death globally. Myocardial infarction is one of the leading causes of death in both developing and developed countries.^        1 ^ Myocardial infarction is characterized by significant irreversible loss of cardiomyocytes, leading to impaired ventricular function. When the heart’s blood flow is interrupted or reduced by a blockage in the coronary artery, cardiomyocytes will be necrotic and will be eventually replaced by scar tissu.^   2 ^ Heart failure occurs at the end of pathological myocardial remodeling due to either ischemic or nonischemic cardiomyopathy.

The routine treatments for people suffering heart attack are balloon angioplasty and coronary artery bypass grafting surgery. However, conventional therapy for heart failure generally does not replace myocardial necrosis with new contractile tissue.^   3 ^ Consequently, new therapeutic approaches have been investigated to decrease myocardial injury and formation of new vessels for ischemic area perfusion. Although heart transplantation is the last therapy option for severe heart failure, shortage of heart donors has limited the application of this therapy. This has led to the development of novel therapeutic approaches, including cellular cardiomyoplasty.^        4 ^ Recently, cell therapy has been performed to prevent myocardial damage via angiogenesis. Overview of the trials indicates that this novel treatment could result in some improvements compared with conventional therapy.^   5 ^

Cell transplantation is a promising treatment strategy for repairing ischemically damaged hearts.^6^^, ^^7^  Over the past decade, various cell types have been proposed as potential useful candidates, including skeletal myoblasts, embryonic cells, hematopoietic cells, and mesenchymal stem cells (MSCs).^   8 ^ Bone marrow contains several types of stem cells with distinct capability of generating cardiomyocytes. MSCs, multipotential stem cells that reside within the bone marrow microenvironment,^   9 ^ can regenerate cardiac muscle by self-renewal and differentiation into cardiomyocytes.^   10 ^ Although bone marrow-derived MSCs have been successfully used experimentally^        11 ^ and clinically,^        12 ^ bone marrow aspiration is a very painful procedure and mostly has been performed under spinal or general anesthesia.^   13 ^

Angiogenesis is a promising new therapy currently under intense investigation. Formation of new vessels can confer better perfusion of the myocardium and subsequently prevent infarct area expansion.^        14 ^ Therapeutic angiogenesis starts with the migration and proliferation of endothelial cells (ECs) as new vascular beds.^        15 ^ It has been reported that endothelial progenitor cells release angiogenic factors like vascular endothelial growth factor and, therefore, augment the cardiac performance, probably by improving regional circulation.^        16 ^ However, ECs are unable to differentiate into cardiomyocytes.^   17 ^

Currently, there is limited information available regarding angiogenesis effects and ventricular function following EC transplantation into myocardial scar tissue.^18^ We hypothesized that MSCs and ECs can promote angiogenesis, one via differentiation into ECs and the other via propagation and proliferation. The objective of the current research project was to compare the effects of EC and MSC transplantation on improving myocardial function using a sheep model of myocardial infarction.

## Methods

All the animal experiments were done in accordance with the National Institute of Health (NIH)’s guideline (Guide for the Care and Use of Laboratory Animals, NIH publication 8523, revised 1996).

The animals included in this study were 18 Iranian ewes (40 ± 5 kg). The animals were clinically healthy and had not been included in any previous experimental study. The animals were housed in standard cages under a protocol approved by the institutional Animal Care and Use Committee. All the animals were fed *ad libitum* with hay and sheep pellets and had free access to water. The animals were assigned to 3 groups: Group I (control, n = 6) underwent diagonal artery ligation with culture media, Group II (MSC, n = 6) underwent diagonal artery ligation with MSC transplantation, and Group III (EC, n = 6) underwent diagonal artery ligation with EC transplantation.

The sheep were anesthetized with intramuscular injections of xylazine (5 mg/kg). Following the iliac crest puncture, a 5–8 mL-sample of bone marrow was promptly aspirated with an 18-gauge needle into a 10-mL syringe containing heparin (3000 units/mL). Also, a piece of saphenous vein (about 5 cm) was harvested for cell culture. ECs, and bone marrow-derived mononuclear cells (BM-MNCs) were isolated according to a method described previously.^19^ Briefly, BM-MNCs and ECs were harvested using a Ficoll gradient centrifugation. Then, the cells were washed with phosphate-buffered saline (PBS), pelleted by centrifugation at 3000 rpm, and resuspended in 5–7 mL of the culture medium of Dulbecco’s modified Eagle’s medium (DMEM; Gibco, U.S.A.) supplemented with 20% fetal bovine serum (FBS; Gibco, U.S.A.). The cells were subsequently seeded into 25T culture flasks and incubated in humidified atmosphere at 37 ˚C with 95% air and 5% CO_2_. The medium was changed every 2 days. During the medium changes, non-adherent mononuclear and red blood cells were discarded. After about 5–7 days, the attached cells grew and developed colonies. The MSCs were passaged twice prior to detachment using trypsin-EDTA (Gibco, U.S.A.). The cells harvested at each passage were used for cell transplantation.

**Figure 1:**
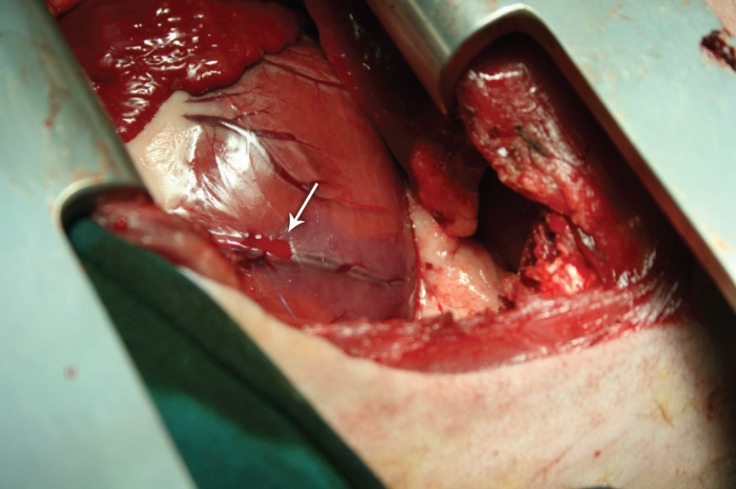
Inducing myocardial infarction in the sheep by ligating (arrow) the 2nd diagonal branch of the left anterior descending artery (left lateral view of the chest).

Experimental myocardial infarction in the sheep was performed according to the procedure described elsewhere.^20^ Briefly, the sheep were nil per os (NPO) 24 hours prior to surgery. Anesthesia was induced with intravenous injections of 5 mg/kg of sodium thiopental and maintained with 2–3 vol% halothane in oxygen. The anaesthetized animals were then orally intubated with a size 7.5 endotracheal tube and mechanically ventilated (Draeger Ventilog 3, Lubeck, Germany) with 100% O_2_, at a tidal volume of 10 mL/kg, and respiratory rate of 12–14/min. After sterile preparation and draping, a left lateral thoracotomy incision (15–20 cm in length) was made through the 4th left intercostal space. After the pericardium was opened, the coronary anatomy was inspected. A 6-0 Prolene™ suture was placed around the 2nd diagonal branch of the left anterior descending coronary artery at an approximately 40% distance from its base (Figure 1). Myocardial ischemia was confirmed by ventricular hypokinesis, cardiac tissue cyanosis, and electrocardiographic (ECG) ST-segment elevation.^20^ Thereafter, 200 µL of culture media (control group), 200 µL of culture media containing 27 × 10^6^ MSCs (MSC group), and 200 µL of culture media containing 27 × 10^6^ ECs (EC group) were injected into the border of the ischemic area at 4 sites after diagonal branch ligation in all the groups separately. Next, the thoracotomy incision was closed (muscles and skin with 2-0 Vicryl™ suture and pericardium with 5-0 Prolene™ suture). For arrhythmia prophylaxis, lidocaine was administered intravenously (2 mg/kg) as a loading bolus dose just before diagonal branch ligation and 15–20 minutes thereafter at 1 mg/kg.^21^ The animals were discharged from the intensive care unit 24 hours after surgery.

Cardiac function was evaluated preoperatively as well as on the 1st postoperative day and in the 2nd postoperative month using transthoracic color Doppler echocardiography, and the left ventricular ejection fraction (LVEF) was calculated. All the measurements were performed by a cardiologist, who was blinded to the treatment group.

The animals were euthanized with approximately 1 g of sodium thiopental. A post-mortem examination of each subject’s heart was performed, and the heart specimens were fixed in 10% buffered neutral formalin, embedded in paraffin, and sectioned at 4 μm. Immunohistochemical staining was performed on the formalin-fixed sections using a panel of antibodies against von Willebrand factor (vWF) and smooth muscle actin (SMA). Vascular density was estimated by the average number of vWF- or SMA-stained blood vessels per high-power field (× 40).

Scanning electron microscopic studies were also performed to monitor qualitative changes in the cardiomyocytes in the area of infarction.

The data analyses were performed using GraphPad software, version 5.01 (GraphPad software Inc., La Jolla, CA, U.S.A.). The data are presented as means ± standard deviations (SDs). The results were compared using the analysis of variance (ANOVA). Differences with p values < 0.05 were considered statistically significant. 

## Results

All the surgical operations were performed without mortality or any major morbidity. The left ventricular ejection fractions (LVEFs) are presented in Table 1. The results of this study show that the EF was initially decreased 1 day after surgery in all the groups. However, 2 months after surgery, significant increases in the mean values of the EF were present in the animals that received MSC or EC transplantation (p value < 0.001 and p value = 0.003, respectively), compared to the control animal group. No statistically significant difference was present between the animals that received MSC transplantation and those that received EC transplantation (p value = 0.230). 

**Table 1:** Pre- and post-operative myocardial ejection fraction (EF) in the sheep that received mesenchymal stem cell (MSC) or endothelial cell (EC) transplantation, compared to the control group*

Group	Preoperative EF (%)	Postoperative EF (%)	
		1 day	2 months
Control	70.33±4.08^a^**	50.17±8.54^a^	48.67±6.80^a^
MSCs	70.67±6.41^a^	48.67±3.33^a^	65.33±4.08^b^
ECs	69.17±7.03^a^	44.00±4.69^a^	62.00±4.86^b^
Effect size	2.99	3.59	1.61

* Data are presented as mean±SD

** Values with similar superscript letters

(a & b) have statistically no significant differences.

**Figure 2:**
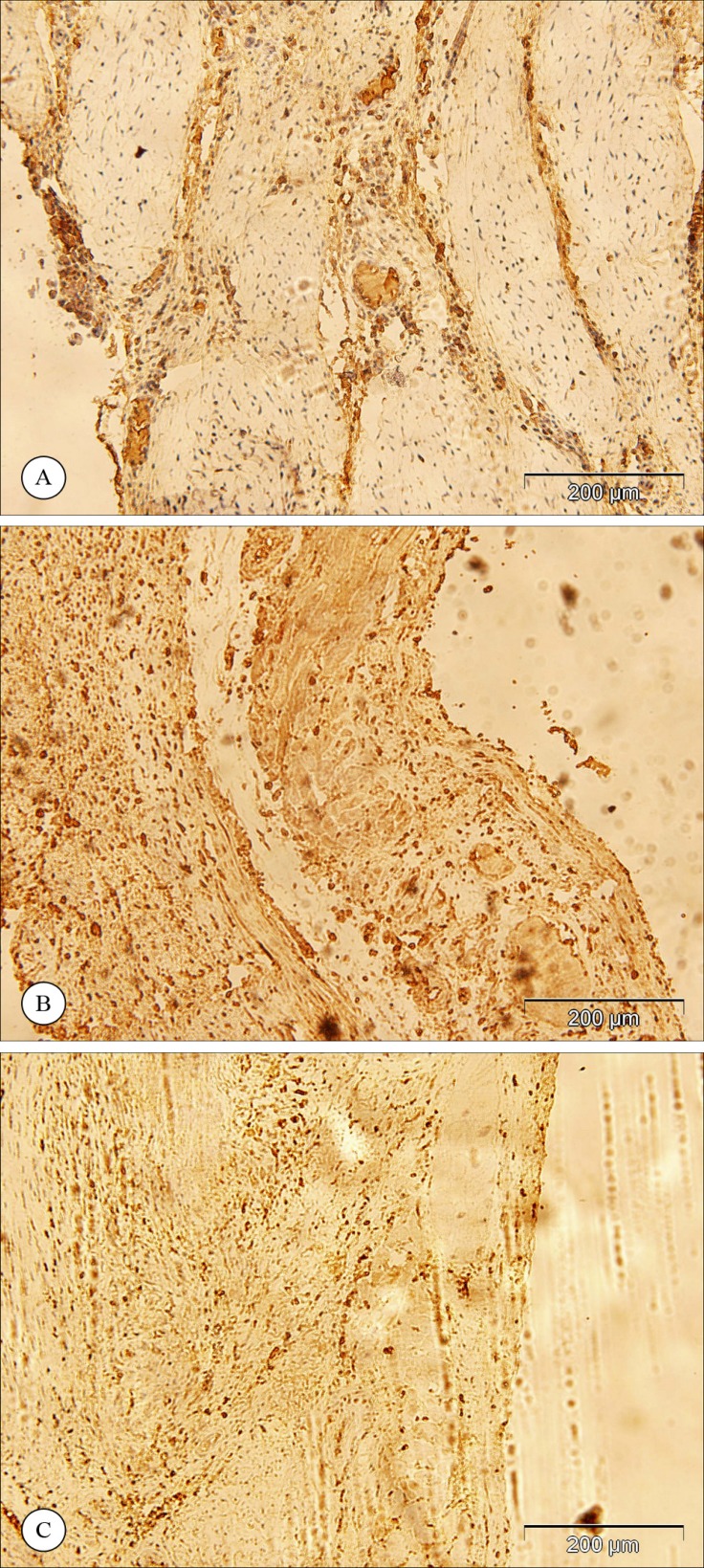
Immunohistochemical staining for von Willebrand factor in the infarct area in the control group (a), in the animals that received mesenchymal stem cells (b) or endothelial cells (c). (Magnification 100X)

**Figure 3:**
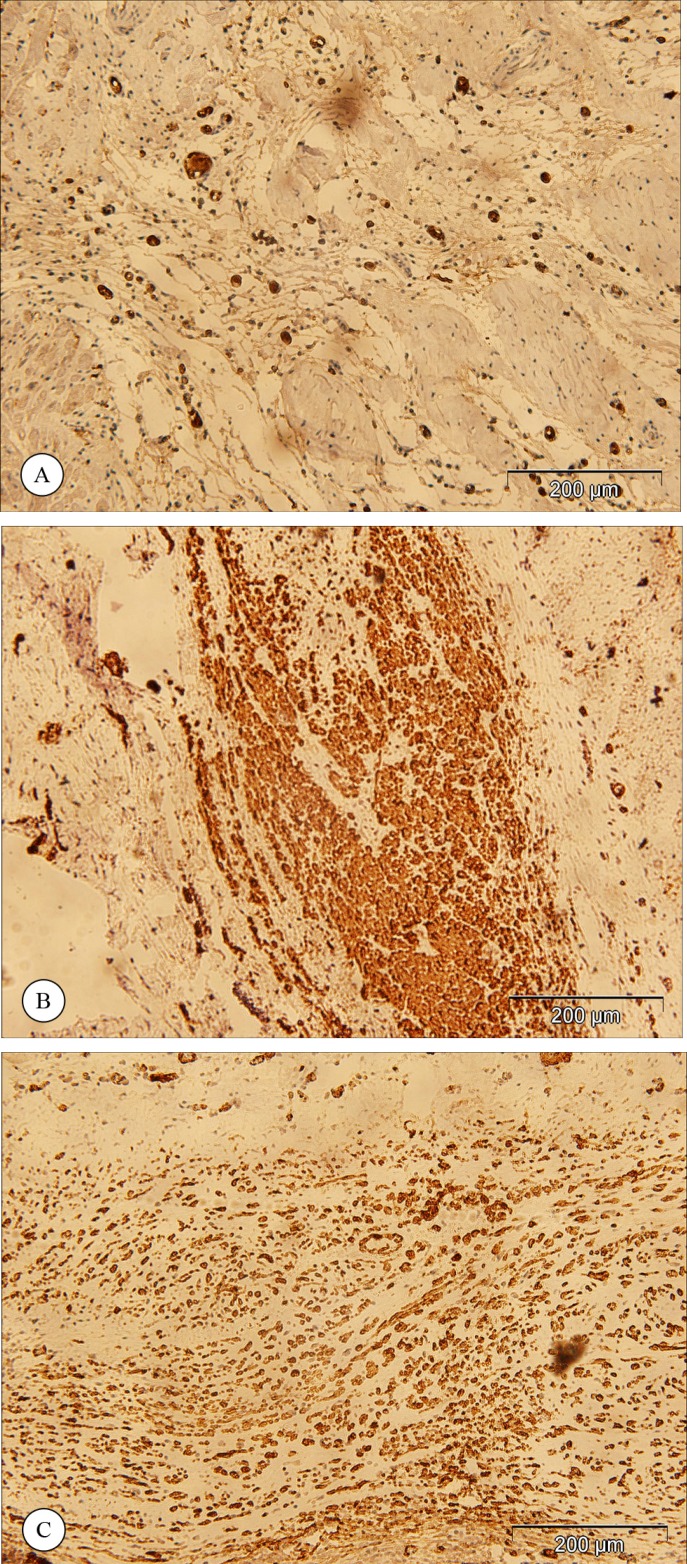
Immunohistochemical staining for smooth muscle actin in the control group (a), in the animals that received mesenchymal stem cells (b), and in the animals that received endothelial cells (c). (Magnification 100X)

Figure 2 and Figure 3 show the results of the immunohistochemical staining for vWF and SMA. The vWF-positive cells were counted in the infarcted area as well as in the border zone (Figure 2), and the SMA-positive cells were counted in the developing vasculature (Figure 3). The results of the counted vWF- or SMA-positive cells are presented in Table 2. The statistical analysis of the number of the vWF-positive cells revealed that capillary density in the infarcted area was significantly greater in the animals that received MSC or EC (p value < 0.001 and p value < 0.001, respectively) transplantation than in the control group. However, no significant difference was present between the MSC and EC groups (p value = 0.865). 

**Table 2:** Vascular density as measured by the number of blood vessels using antibodies against von Willebrand factor (vWF) and smooth muscle actin (SMA) in the sheep 2 months after mesenchymal stem cell (MSC) or endothelial cell (EC) transplantation*

Group	vWF		SMA
	Infarct Area	Border Zone	
Control	23.28±1.24^a^**	29.60±5.87^a^	27.73±8.14^a^
MSCs	60.91±20.50^b^	79.45±18.72^b^	103.59±66.16^b^
ECs	63.02±18.75^b^	78.00±13.95^b^	89.90±42.77^b^
Effect size	2.99	3.59	1.61

* Data are presented as mean number of vessels per high power field of microscope±SD

** Values with similar superscript letters

(a & b) have statistically no significant differences.

The results of vascular density in the border zone were similar to those in the infarcted area. The differences between the control group and the MSC or EC groups were statistically significant (p value < 0.001). No significant difference was present between the MSC and EC groups (p value = 0.881). Furthermore, the number of SMA-positive arterioles was statistically higher in the MSC (p value = 0.023) and EC (p value = 0.006) groups than in the control animals. No statistically significant difference was noted between the MSC and EC groups (p value = 0.684). 

In the electron microscopic studies of the infracted areas, cardiomyocytes in variable stages of development were seen in the border zone in both MSC and EC groups (data not shown).

## Discussion

Several animal studies have been performed using MSC transplantation after myocardial infarction and demonstrated positive effects on left ventricular (LV) function, reduction of the infarct size, and reduction of mortality rate. Shinji Tomita et al.^22^ in 1999 used autologous bone marrow cells transplanted into ventricular cryoinjured scar tissue. They differentiated these cells to cardiomyocytes and observed angiogenesis and improved cardiac function. Dai et al.^23^ showed that allogeneic MSC transplantation in the post-infarcted rat myocardium was able to transiently improve global LV function at 4 weeks possibly due to an early paracrine effect. In a canine chronic ischemia model, Silva et al.^24^ reported that MSC differentiation into endothelial and smooth muscle cells was able to increase vascularity and improve cardiac function. Grauss et al.^25^ used human MSCs in immunocompromised non-obese diabetic/ susceptible insulin dependent (NOD/SCID) mice and showed improved LV function and limited LV remodeling. Miyahara et al.^26^ used monolayered MSC sheets in infarcted rat hearts. They observed undifferentiated cells, newly formed vasculature, and few cardiomyocytes in the engrafted sheet. The monolayered MSCs improved cardiac function in rats with myocardial infarction. 

MSCs can be easily cultured and they can remain multipotent. Moreover, they are highly proliferative and can differentiate into many cell types, especially smooth muscle and ECs; however, they have limited potential to differentiate into cardiomyocytes.^27^ The differentiation of these cells is age-dependent and aged cells may show low expansion *in vitro*. Also, obtaining these cells requires bone marrow aspiration, which is an inconvenient method for the patients. Furthermore, these cells may have some side effects such as fibrosis and calcification.^28^ It has been shown that the transplantation of bone marrow-derived MSCs with and without co-culture with myocardial cells reduces the infarct size in ischemic rat hearts.^29^ Myocardial injection of human STRO-1 mesenchymal progenitors, in an athymic rat model of acute myocardial infarction, enhanced vascular density, induced neovascularization, and improved myocardial ejection fraction (EF).^30^

In contrast with MSCs, EC transplantation for improving myocardial function has been rarely employed. EC transplantation is less time-consuming and is a relatively cost-effective procedure. ECs can be easily obtained from a piece of vessel. Although these cells are also age-dependent, EC transplantation has no side effects. ECs never differentiate into bone or any other undesirable tissues. Following the injection of ECs, only angiogenesis and improved cardiac function via formation of new vessels are expected. In a rat model of cardiac injury, EC transplantation into a myocardial scar stimulated angiogenesis and increased regional perfusion in myocardial scar tissue without any positive effect on global function.^18^

Using autologous mature EC transplantation could become a useful alternative to other cell therapy. ECs induce a wide capillary network; however, their transplantation may not lead to sufficient functional vasculature formation. A reduced EF was found to be one of the major determinants of cardiac mortality. The results of our study showed that the transplantation of both MSCs and ECs was able to improve cardiac function and induce angiogenesis. MSCs can differentiate into ECs and hence prevent an increase in the ischemic area. Apropos the effect size (Table 1 and Table 2), as was expected, where the p values were significant, our study had a good power and where the p values were not significant, there was a small effect size.

The electron microscopic studies showed that the ECs had also differentiated into cardiomyocytes. This can prevent a drop in the EF via decreasing the infracted area. The appearance of developing cardiomyocytes in the EC group can be at least partially due to a better perfusion of the heart and the presence of appropriate environment for the native stem cells to differentiate into cardiomyocytes.

In light of the results of this work, we can suggest the use of adult ECs instead of stem cells obtained from the very invasive method of bone marrow aspiration. Only a part of vein can be harvested to obtain adult ECs. 

## Conclusion

The results of this study demonstrated that EC transplantation, similar to MSC transplantation, was able to improve cardiac function and angiogenesis. Further studies on the use of ECs for myocardial regeneration are required to arrive at a firm conclusion on the usefulness of these cells. 

## Notes:

***This paper should be cited as:*** Rabbani S, Soleimani M, Sahebjam M, Imani M, Nassiri SM, Atashi A, Daliri Joupari M, Ghiaseddin A, Latifpour M, Ahmadi Tafti SH. Effects of Endothelial and Mesenchymal Stem Cells on Improving Myocardial Function in a Sheep Animal Model. J Teh Univ Heart Ctr 2017;12(2):65-71.
